# Management of lower urinary tract symptoms before prostate cancer radiotherapy: a call for innovative strategies

**DOI:** 10.1007/s00345-026-06427-9

**Published:** 2026-04-30

**Authors:** Jennifer Le Guevelou, Julien Anract, Charles Dariane, Matthijs Scheltema, Rui Bernardino, Lorenzo Bianchi, Veeru Kasivisvanathan, Mattia Sibona, Andrea Mari, Marc Sapoval, Marcin Miszczyk, Pawel Rajwa, Tom Boeken, Giancarlo Marra

**Affiliations:** 1 University of medicine François Rabelais, Tours, France; 2 Department of radiotherapy, CHRU Tours, Boulevard Tonnelé, Tours, France; 3 Department of Urology, AP-HP, Hôpital Cochin, Université Paris Cité, Paris, France; 4 Department of Urology, Hôpital Européen Georges Pompidou, AP-AP, Paris, France; 5 U1151 Inserm-INEM, Paris-Cité University, Paris, France; 6 St. Antonius Hospital, Utrecht, The Netherlands; 7 Glickman Urological Institute, Cleveland Clinic Foundation, Cleveland, OH USA; 8 Division of Urology, IRCCS Azienza Ospedaliero-Universitaria di Bologna, Bologna, Italy; 9 Università degli studi di Bologna, Bologna, Italy; 10 Centre for Urology Imaging, Prostate, AI and Surgical Studies (COMPASS) Research Group, Division of Surgery and Interventional Science, University College London, London, UK; 11 Città della Salute e della Scienza e Università degli Studi di Torino, Torino, Italy; 12 Oncologic Minimally Invasive Urology and Andrology Unit, Department of Experimental and Clinical Medicine, Careggi Hospital, University of Florence, Florence, 50121 Italy; 13 Department of Vascular and Oncological Interventional Radiology, Assistance Publique-Hôpitaux de Paris, Hôpital Européen Georges Pompidou, Paris, 75015 France; 14 Université Paris Cité, PARCC - INSERM Unité-970, Paris, 75015 France; 15 Department of Urology, Comprehensive Cancer Center, Medical University of Vienna, Vienna, Austria; 16 Collegium Medicum - Faculty of Medicine, WSB University, Dąbrowa Górnicza, Poland; 17 Department of Urology, Medical University of Silesia, Zabrze, Poland

**Keywords:** Prostate cancer, Lower urinary tract symptoms, Radiotherapy, Minimally-invasive surgical treatment, Transurethral resection of the prostate

## Abstract

Benign prostatic hyperplasia (BPH) is the main cause of lower urinary tract symptoms (LUTS) in aging men. As the incidence of prostate cancer (PCa) increases with age, coexistence of BPO and PCa is a frequent issue. Radical prostatectomy might be considered as the best option for patients presenting with severe obstructive symptoms, allowing to simultaneously treat both diseases. However, not all patients are eligible or prefer surgery, therefore a large number of patients are referred to receive PCa radiotherapy (RT). Preexisting LUTS represent a well-known risk factor to develop severe genito-urinary (GU) toxicity. While improving urinary function before starting irradiation appears to be crucial, the development of new minimally invasive surgical therapies (MIST) for BPH creates new opportunities for treatment personalization in RT patients with LUTS.

## Introduction

Benign prostatic hyperplasia (BPH) can cause benign prostatic enlargement (BPE) which can itself result in benign prostatic obstruction (BPO) [1]. BPO is the main cause of lower urinary tract symptoms (LUTS) in aging men. As the incidence of prostate cancer (PCa) increases with age [2], coexistence of BPO and PCa is a frequent issue. Radical prostatectomy might be considered as the best option for patients presenting with severe obstructive symptoms, allowing to simultaneously treat both diseases. However, not all patients are eligible or prefer surgery, therefore a large number of patients are referred to receive PCa radiotherapy (RT). According to European Association of Urology (EAU) guidelines, LUTS severity and risk stratification should rely on validated symptom scores and post-void residual (PVR) measurements. Urodynamic studies play a selective role in confirming BPO due to hyperplasia, in case of diagnostic uncertainty [3]. This step is particularly relevant in patients planned for RT, as preexisting LUTS (but not asymptomatic BPE) represent a well-known risk factor to develop severe genito-urinary (GU) toxicity [4–7]. While improving urinary function before starting irradiation appears to be crucial [8], the development of new minimally invasive surgical therapies (MIST) for BPH creates new opportunities for treatment personalization in RT patients with LUTS.

**Fig. 1:**
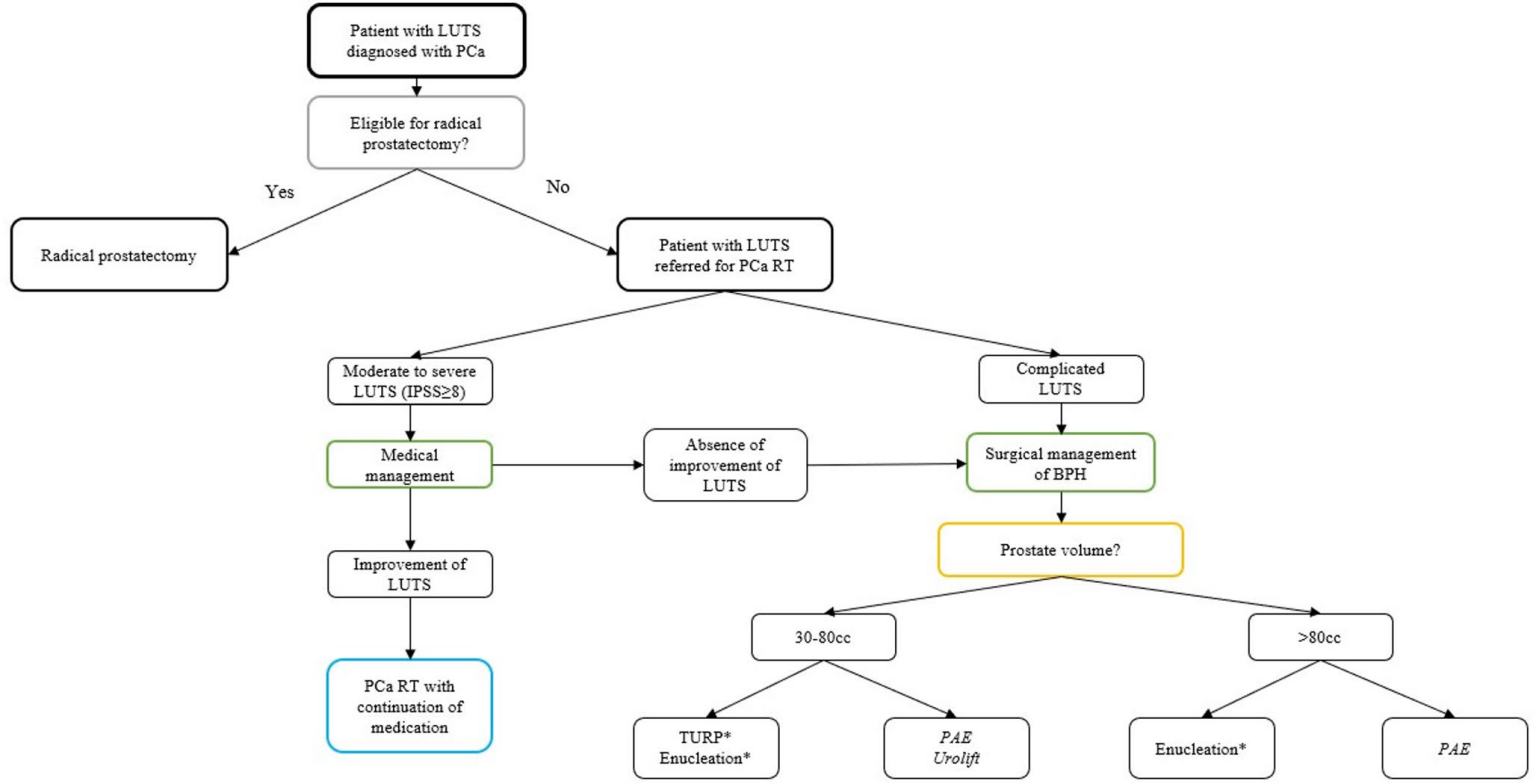
proposed algorithm for the management of LUTS in patients referred for prostate cancer radiotherapy. *LUTS* lower urinary tract symptoms, *PCa* prostate cancer, *RT* radiotherapy, *BPH* benign prostatic hyperplasia, *PAE* prostate artery embolization, *TURP* transurethral resection of the prostate.*considered as SOC

Medical interventions represent the first-line of treatment for men with moderate-to-severe LUTS [3], and should represent the initial step in most patients planned for PCa RT. Guideline-recommended treatments, including alpha-blockers, phosphodiesterase type 5 inhibitors or combination therapy [3] may provide adequate symptom control and reduce the risk of treatment-related urinary complications in a substantial proportion of patients. Surgical strategies should be considered in the event of ineffective drug treatment, patient-reported intolerance or unwillingness to long-term pharmacological treatments, or BPO-related urinary tract complications (Fig. 1) [9].

Simple prostatectomy was one of the earliest introduced treatment options for the management of BPH, and is still considered as a standard-of-care, particularly when endoscopic enucleation is not available. This strategy allows for a complete excision of the adenoma, and is associated with a significant improvement in IPSS (up to 23 points) [10, 11]. Although this technique is associated with an exceedingly low reintervention rate at 5 years, it is associated with a morbidity that can include both incontinence and urethral stenosis, which may be a concern in patients that are planned to receive PCa RT. Also, it is associated with a significant risk of transfusion, making the recovery longer and thus delaying further treatments. Transurethral resection of the prostate (TURP) has been effectively used for decades but can also result in urethral stenosis (up to 20% [12]), incontinence (1.5% [13]) and urinary tract infection (4% [14]) [15, 16]. A history of TURP has been reported to be a risk-factor of severe GU toxicity following dose-escalated PCa RT in several prospective trials [4, 17–20]. This excess of risk has also been reported after prostate stereotactic body radiotherapy (SBRT), driven by an increased risk of hematuria [21–24]. Although the time between TURP and RT has not been formally identified as a factor influencing the occurrence of severe toxicity, the GETUG recommends a 3-month interval between the two procedures [25]. Overall, it remains unknown whether or not TURP per se represents a risk-factor of severe GU toxicity or if it represents a surrogate for persistent LUTS despite surgical treatment [26]. In the past decade, endoscopic enucleation of the prostate (EEP) emerged as an alternative to TURP. EEP is a guideline-supported option that can be performed regardless of prostate size. It provides the advantages of being associated with lower retreatment rates compared with TURP [27] (Table 1). However, EEP and specifically HoLEP was associated to a significant risk for transient urgency incontinence (34%) occurring in the firsts three months [10, 28], highlighting the need for a minimum time-interval between EEP and PCa RT. Scarce data are available on patients with a previous history of EEP that receive PCa RT [29, 30], and to date no study compared outcomes with TURP or EEP before PCa RT.

**Table 1:** Advantages and limitations of surgical and minimally-invasive surgical treatment before prostate cancer radiotherapy

Technique	Prostate volume	Feasible in case of a median lobe	Tolerability	Available data before PCa RT	Ablative or non-ablative techniques *	Reduction in IPSS at 12 months **	Time interval between procedure and PCa RT***	Reoperation rates**
Simple prostatectomy	> 80cm^3^	Yes	Hospital stays (3–5 days) High rates of blood transfusion (7–14%) Requires general anesthesia	Limited amount of data	Ablative	15–20 points	Minimum 3 months	1.3% at 1 year 4.4% at 5 years
TURP	30-80cm^3^	Yes	Hospital stays (2–3 days) Low rates of blood transfusion (2–8%) Requires general anesthesia	Large amount of data	Ablative	12–16 points	Minimum 3 months	4.0% at 1 year 7.7% at 5 years
EEP	> 30cm^3^	Yes	Hospital stays (1–2 days) Low rates of blood transfusion (0–2%) Requires general anesthesia	Limited amount of data (< 10 studies)	Ablative	15–20 points	Minimum 3 months	2.4% at 1 year 6.6% at 5 years
PAE	> 40cm^3^	Yes	Hospital stays (0–1 days) No blood transfusion (0%) Can be performed without general anesthesia	Limited amount of data (< 10 studies)	Ablative	13–16 points	Minimum 6 weeks Ideally 3 months (to obtain significant prostate size reduction)	12.2% at 1 year 23.8% at 5 years
Intraprostatic implants	< 30 cc to 80cm^3^	Relative contraindication	Hospital stays (0–1 days) No blood transfusion (0%) Can be performed without general anesthesia	Limited amount of data (< 10 studies)	Non-ablative	9–12 points	Theoretically, no minimum time-interval	3.0% at 1 year 28.9% at 5 years
Convective water vapour energy ablation	30–80 cm^3^	Yes	Hospital stays (0–1 days) No blood transfusion (0%) Can be performed without general anesthesia	No data	Ablative	10–12 points	Minimum 6 weeks	3.0% at 1 year 4.4% at 5 years
Aquablation	30–80 cm^3^	Yes	Hospital stays (1-1.5 days) Low rates of blood transfusion (2–4%) Required general anesthesia	No data	Ablative	14–17 points	Minimum 6 weeks	0.7% at 1 year 4.4%-6% at 5 years

*TURP* transurethral resection of the prostate, *EEP* endoscopic enucleation of the prostate, *PAE* prostate artery embolization, *PCa* prostate cancer, *RT* radiotherapy

*Ablative or non-ablative techniques as described in the eau guidelines available at: HTTPS://D56BOCHLUXQNZ.CLOUDFRONT.NET/DOCUMENTS/FULL-GUIDELINE/EAU-GUIDELINES-ON-NON-NEUROGENIC-MALE-LUTS-2024.PDF

** After procedure only and outside of the context of prostate cancer radiotherapy

***According to experts’ opinion and in the absence of guidelines or level A evidence

MISTs are increasingly being tested in men with BPH as they demonstrated lower rates of complications than surgical strategies. While intraprostatic implants (IPI), prostatic artery embolization (PAE), water vapor thermal therapy and Aquablation have recently been implemented within guidelines, they could represent an alternative to both TURP and EEP before PCa RT.

PAE consists in a bilateral embolization of prostatic arteries, that induces an ischemic shrinkage of the prostate gland. Among the key aspect of this strategy lies its feasibility in patients with large prostate volumes (over 80cm^3^). The PARTEM phase III trial recently demonstrated the superiority of this approach compared with a medical treatment (dutasteride and tamsulosine), in patients with LUTS resisting to alpha-blockers, with a 10 point-reduction in IPSS observed at 9 months (compared with 5.7, *p* = 0.0008) [31]. Urinary complications were usually minor and included pollakiuria and transient dysuria. However, these results cannot be extrapolated to PCa patients, and currently, a limited number of studies reported outcomes of PAE performed in men planned to receive PCa RT. Overall, PAE performed in PCa patients was associated with an IPSS reduction ranging between 11 and 14 points [32, 33]. After PCa RT, only a modest impairment in urinary quality of life was observed compared with immediate outcomes after PAE (IPSS increase reaching 3 points at 6 months, with no further increase after 6 months). PAE as a neoadjuvant approach in men planned to receive PCa RT is however associated with potential limitations, and especially its high rates of invasive retreatment, reaching 12.5% at 9 months and up to 23.8% at 5 years [34]. Considering that surgical deobstructive approaches performed after PCa RT are associated with a high-risk of GU toxicity (i.e. incontinence and/or urethral calcification-strictures [8]), reoperation rates should be considered as a crucial endpoint in this neoadjuvant setting. Also, the efficacy of PAE in reducing urodynamic bladder obstruction is questionable. Therefore, it is unclear if they could be suitable for neoadjuvant treatment in patients with severe obstruction [35]. On a radiobiological level, concerns may be raised due to the increase in hypoxia within prostatic tissues following PAE, as pretreatment hypoxia recently emerged as a common feature of poor-prognosis PCa [36]. However, it was hypothesized that PAE would induce hypoxia within the central portion of the prostate gland but not within the peripheral zone [37], where the majority of PCa forms. Last but not least, and similarly to what is usually advocated if a TURP is performed before PCa RT, a minimal time-interval between PAE and PCa RT might be necessary in order to allow for complete tissue healing, as a shorter interval between PAE and PCa RT has been suggested to be associated with an increased risk to develop late GU toxicity (< 15 weeks vs. ≥ 15 weeks HR 3.56, 95% CI 1.13–11.24, *p* = 0.02) [32]. Also, a minimal time-interval is required to allow for an optimal prostate volume reduction. Although PAE appears promising and is included in the EAU guidelines with caution [3], it should currently be considered an experimental option in the neoadjuvant RT setting, as it is not recommended as standard first-line therapy, lacks oncological validation, and carries a non-negligible risk of retreatment, which may have important clinical implications once RT has been delivered.

IPI allows to pull the lateral lobes of the prostate gland away from the urethral lumen, thereby mechanically opening the prostatic urethra without requiring any tissue resection. UroLift has been shown to provide a rapid (2 weeks) improvement in LUTS [16] which can be of interest in the setting of PCa as it would allow to plan RT without further delay. Also, this strategy is non-ablative and thereby preserves the integrity of urinary structures such as the intraprostatic urethra and the bladder neck. However, the placement of an intraprostatic device followed by RT might cause concerns regarding the development of inflammation and fibrosis within the intraprostatic urethra. IPI are associated with smaller IPSS gains compared with surgical ablative strategies such as TURP and EEP beyond 6 to 12 months, and surgical retreatment rates raises up to 13.6% at 5 years [38] which might raise concerns of further GU toxicity in patients that receive PCa RT. Last but not least, this strategy is usually not recommended in patients with prostate volume over 80cm^3^ as well as patients with presence of a median lobe. Prospective trials are currently testing the placement of IPI before PCa RT. The CO-STAR phase II trial (NCT05840549) randomizes either IPI or TURP in patients with LUTS that are planned to receive PCa RT. The primary aim of this trial is to assess the feasibility of IPI in this disease setting. Also, a phase I trial (NCT05311527) was recently led with the aim to assess IPI’s safety performed before stereotactic ablative body radiotherapy (SABR).

The landscape of the MISTs includes also other techniques, that were quite widely studied in a variety of settings, but not as a neoadjuvant therapy before PCa. Water vapor thermal therapy offers a significant improvement in IPSS (10 to 12 points) [39], can be performed without the requirement of general anesthesia, and is associated with a low morbidity profile. Aquablation also represent a recently validated alternative, associated with a significant IPSS gain at 12 months (14–17 points) [40], and a low rate of retreatment at 5 years [41]. However, these techniques being recent, no data are available regarding their feasibility before PCa RT.

To conclude, medical therapies still represent the first line of treatment in men presenting with LUTS and planned to receive PCa. Surgical strategies should be considered in the event of ineffective drug treatment, patient-reported intolerance or unwillingness to long-term pharmacological treatments, or BPO-related urinary tract complications. MIST would provide the advantage of a higher tolerability, however might be associated with a lower IPSS benefit and a higher risk of surgical retreatment. MIST should currently be considered an experimental option in the neoadjuvant RT setting, as it is not recommended as standard first-line therapy.

## Data Availability

No datasets were generated or analysed during the current study.
